# A Novel Restorative Pulmonary Valve Conduit: Early Outcomes of Two Clinical Trials

**DOI:** 10.3389/fcvm.2020.583360

**Published:** 2021-03-04

**Authors:** David L. Morales, Cynthia Herrington, Emile A. Bacha, Victor O. Morell, Zsolt Prodán, Tomasz Mroczek, Sivakumar Sivalingam, Martijn Cox, Gerardus Bennink, Federico M. Asch

**Affiliations:** ^1^Department of Cardiothoracic Surgery, Cincinnati Children's Hospital Medical Center, Cincinnati, OH, United States; ^2^Division of Cardiothoracic Surgery, Children's Hospital Los Angeles, Los Angeles, CA, United States; ^3^Division of CardioThoracic Surgery, Columbia University Medical Center, New York, NY, United States; ^4^Division of Pediatric Cardiothoracic Surgery, Children's Hospital of Pittsburgh, University of Pittsburgh Medical Center, Pittsburgh, PA, United States; ^5^Department of Pediatric Heart Surgery, Gottsegen György Hungarian Institute of Cardiology, Budapest, Hungary; ^6^Department of Pediatric Cardiac Surgery, Jagiellonian University, Krakow, Poland; ^7^Department of Cardiothoracic Surgery, Institut Jantung Negara, Kuala Lumpur, Malaysia; ^8^Xeltis BV, Eindhoven, Netherlands; ^9^Department of Cardiothoracic Surgery, University Hospital of Cologne, Cologne, Germany; ^10^Cardiovascular Core Laboratories, MedStar Health Research Institute, Washington, DC, United States

**Keywords:** pulmonary valved conduit, endogenous tissue restoration, clinical trial, RVOT reconstruction, biocompatibility

## Abstract

**Objectives:** We report the first use of a biorestorative valved conduit (Xeltis pulmonary valve–XPV) in children. Based on early follow-up data the valve design was modified; we report on the comparative performance of the two designs at 12 months post-implantation.

**Methods:** Twelve children (six male) median age 5 (2 to 12) years and weight 17 (10 to 43) kg, had implantation of the first XPV valve design (XPV-1, group 1; 16 mm (*n* = 5), and 18 mm (*n* = 7). All had had previous surgery. Based on XPV performance at 12 months, the leaflet design was modified and an additional six children (five male) with complex malformations, median age 5 (3 to 9) years, and weight 21 (14 to 29) kg underwent implantation of the new XPV (XPV-2, group 2; 18 mm in all). For both subgroups, the 12 month clinical and echocardiographic outcomes were compared.

**Results:** All patients in both groups have completed 12 months of follow-up. All are in NYHA functional class I. Seventeen of the 18 conduits have shown no evidence of progressive stenosis, dilation or aneurysm formation. Residual gradients of >40 mm Hg were observed in three patients in group 1 due to kinking of the conduit (*n* = 1), and peripheral stenosis of the branch pulmonary arteries (*n* = 2). In group 2, one patient developed rapidly progressive stenosis of the proximal conduit anastomosis, requiring conduit replacement. Five patients in group 1 developed severe pulmonary valve regurgitation (PI) due to prolapse of valve leaflet. In contrast, only one patient in group 2 developed more than mild PI at 12 months, which was not related to leaflet prolapse.

**Conclusions:** The XPV, a biorestorative valved conduit, demonstrated promising early clinical outcomes in humans with 17 of 18 patients being free of reintervention at 1 year. Early onset PI seen in the XPV-1 version seems to have been corrected in the XPV-2, which has led to the approval of an FDA clinical trial.

**Clinical Trial Registration:** www.ClinicalTrials.gov, identifier: NCT02700100 and NCT03022708.

## Introduction

Reconstruction of the right ventricular outflow tract (RVOT) with a valved conduit is required for a variety of structural cardiac malformations, and is one of the commonest procedures in pediatric heart surgery. Currently available valved autografts and allografts have a limited lifespan, and are constrained by their inability to grow with the somatic growth of the patient, necessitating repeated surgery (1). Some of them like homografts also do not meet the requirements of ready availability and low antigenicity. Providing a truly restorative alternative for this patient group has been a long sought-after goal in the field of cardiovascular tissue engineering, pioneered by the work of Shinoka et al. (2), who were the first to implant an *in vitro* tissue-engineered leaflet replacement in a lamb model, later continued in the work of Hoerstrup et al. using *in vitro* (3). In both approaches, a fast-degrading polymeric scaffold is seeded *in vitro* with cells, followed by bioreactor culture to initiate formation of autologous tissue. More recently, several research groups have been investigating the addition of a decellularization step at the end of this process, which reduces issues observed with leaflet retraction due to presence of overly active cells and may open doors to non-autologous (allogeneic) approaches. Particularly worthwhile is the work by Emmert et al. (4), who showed 1-year functionality in sheep for a pulmonary valve based on this concept and Reimer et al. (5), who used a fibrin-based decellularized *in vitro* tissue engineered approach to show growth potential in a pulmonary valve model. To date, these applications have yet to demonstrate clinical feasibility. Alternative approaches include the use of decellularized homografts and xenografts as a template for *in situ* tissue restoration. The xenograft has been approached with caution after the porcine Synergraft experience, where a clinical trial had to be aborted prematurely due to an immune response to residual porcine cells (6). Decellularized homografts have been applied with some success in clinic (7), although this approach does not resolve the issue of shortage of human donor valves. An alternative approach therefore would be to use an off-the-shelf fully synthetic implant. Currently used synthetic implants (e.g., ePTFE, Dacron) are typically design to be biostable and inert with minimal tissue response. While commonplace for larger diameter vessels, synthetics face patency limitation for small diameter (<4 mm) vessels. Previous attempts with synthetic heart valves have faced limited success in clinic, although some new clinical trials have been initiated recently e.g., using biostable polyurethane (8) or extra-thin ePTFE (9). The ultimate approach would combine the benefit of off-the-shelf synthetic materials with the potential to restore functional natural tissue upon implantation. This approach is an active topic of academic research and may vary in whether or not cells and/or bioactive factors are included to guide tissue outcome (10). Previously we reported favorable up to 12 months outcomes in sheep for an off-the-shelf bioabsorbable and restorative pulmonary valved conduit, which is devoid of any bioactive factors or cells (11). The current investigation compares the 12 month outcomes of two small clinical studies with two consecutive designs of the Xeltis pulmonary valved conduit (XPV).

## Methods

### Ethical Approval

Regulatory Authority approvals (or notification, according to national regulation) were obtained from each country. The studies were aligned to Good Clinical Practice in compliance with the “Declaration of Helsinki” (12, 13). Informed consent was obtained from the parents for all patients prior to the procedure (ClinicalTrials.gov No: NCT02700100 and NCT03022708).

### The Xeltis Pulmonary Valve Conduit

**Figure 1:**
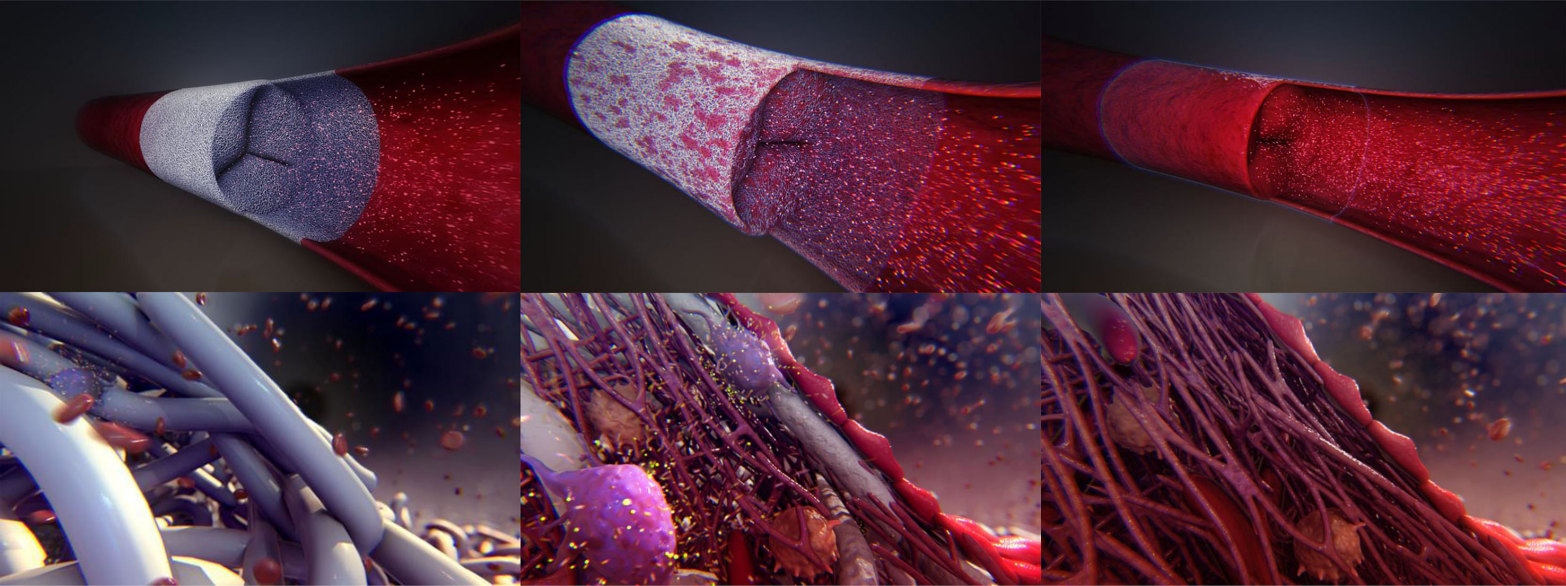
Artistic impression of intended endogenous tissue restoration process at implant **(Top)** and microstructural level **(Bottom)**. The porous implant is functional upon placement and gets permeated with blood components including red and white blood cells and platelets. Subsequently, macrophages are recruited as part of the host-material response. Macrophages governing tissue remodeling by recruiting other cells including (myo)fibroblasts and endothelials cells and govern implant absorption by secreting oxygen radicals, eventually resulting in a functionally restored natural implant.

The Xeltis pulmonary valve conduit (XPV) is fully synthetic, seamless, flexible, highly porous, and bioabsorbable. It makes use of the RestoreX™ polymer platform, which building blocks are based on supramolecular 2-ureido-4[1H]-pyrimidone (UPy) (14). The conduit wall consists of poly-caprolactone-based UPy, and the leaflets are made of poly-carbonate-based UPy, which provides flexibility for leaflet motion. The implanted graft material is designed to attract patient's own cells and proteins, and trigger a set of events that lead to endogenous tissue restoration (Figure 1). As the graft resorbs progressively after implantation, all components of native tissue invade the graft and the leaflets, and develop and organize into natural tissue (11). Based on animal histological data it takes up to 12 months for the leaflets to be functionally replaced with native tissue. As proof of concept, an initial clinical trial involving valveless conduit grafts was successfully conducted in a small series of patients undergoing completion of the Fontan operation by use of an extracardiac conduit (15). This concept has now been extended to the addition of a three leaflet valve within the conduit, for endogenous restoration of the RVOT.

Although XPV conduits of any desired diameter and degree of leaflet coaptation can be manufactured with the available technology, the XPVs used in both trials had a diameter of either 16 or 18 mm, and an initial conduit length of 8 cm.

### Patients

Twelve patients (six male) underwent open heart surgery for XPV implantation with the initial clinical prototype at one of three centers worldwide (group 1). Valves were placed in the normal fashion for a right ventricle to pulmonary artery conduit and no change in technique was reported by surgeons. Their median age was 5 years (range 2 to 12), median weight 17 kg (10 to 43). Individual diagnoses were tetralogy of Fallot (*n* = 4), pulmonary atresia with ventricular septal defect (*n* = 4), common arterial trunk (*n* = 3), and transposition of the great arteries with ventricular septal defect and pulmonary stenosis (*n* = 1). All of them had undergone prior open heart surgery to repair the primary defect; six of them had received a previous valved conduit, which was replaced by the XPV; the others had had a transannular patch. Following evaluation of their 12 month post-surgery outcome (completed in March 2018), modifications in the valve design were undertaken, and a second cohort of patients (*n* = 6, 5 male, group 2) underwent open heart surgery for XPV implantation between March and October 2018. No change in technique was reported. These procedures were performed at one of four North American centers. Their median age was 5 years (3 to 9 years), and median weight 21 kg (14 to 29). Individual diagnoses were tetralogy of Fallot (*n* = 1), pulmonary atresia with ventricular septal defect (*n* = 1), transposition of the great arteries with ventricular septal defect and pulmonary stenosis (*n* = 1; and common arterial trunk (*n* = 1). In two children the XPV was implanted in the RVOT as part of the Ross procedure (both patients had previously undergone aortic valve interventions).

### Surgery

In group 1, five children had a 16 mm diameter conduit implanted, and seven children received an 18 mm diameter conduit. All patients in group 2 received an 18 mm diameter conduit. In three patients in group 1, the distal pulmonary arteries were concomitantly enlarged using a pericardial patch. Transesophageal echocardiography imaging was used intraoperatively in all subjects.

### Modification of the XPV Conduit Design Prior to Implantation in Group 2

The technology described in this manuscript relies on a gradual transitioning of functionality from the original implant toward newly formed patient-own tissue. Early onset of significant PI at 12 months in group 1 (see Results, below) suggested that this balance may not have been adequate for all patients, which prompted technical modifications of the valved conduit. Retrospective analysis of the first generation XPV conduits revealed an inhomogeneity in leaflet thickness distribution, leaflets being thinnest near the commissures. Computational modeling of the diastolic phase of the cardiac cycle, i.e., when pressure over valve is highest, revealed that these thin areas were correlated with areas of highest leaflet tensile stress concentration, and bench testing at elevated pressures and frequencies revealed commissural tear as the most typical failure mode. The second generation design was therefore optimized toward increasing thickness in these areas, resulting in a more homogenous thickness distribution together with a slight increase in average leaflet thickness. Further increasing commissural thickness faces limitations due to reduced leaflet flexibility. Bench testing of the second generation design revealed a significant increase in fatigue resistance. *In vivo*, this is anticipated to provide more time for the newly formed tissue to build strength and take over functionality, which may help improve clinical outcome.

### Follow-Up

All children were evaluated clinically, and by serial echocardiography at hospital discharge, as well as at 6 weeks, three, six, nine, and 12 months, respectively. Haemodynamic performance of the conduit was assessed by cross sectional echocardiography. Stenosis was assessed in the parasternal short axis view, using the continuous wave Doppler technique to measure the peak velocity of flow through the conduit. PI was qualitatively assessed using previously published criteria combining color flow and pulsed Doppler echocardiography (16). Regurgitation was classified as none, trivial or trace, mild, moderate or severe. All echocardiographic studies were independently analyzed by a core laboratory, which was unaware of the individual patient's clinical or demographic data.

### Data Collection and Statistical Analysis

All peri- and postoperative data were collected prospectively according to the detailed clinical study protocol. Data are described as mean with standard deviation (SD) if continuous, and as counts and percent if categorical. Minitab 19 (Minitab, State College, PA, USA) software was used for statistical analysis. The Anderson—Darling normality test was used to assess whether the data were normally distributed. In case of normally distributed data, the *t*-test was used; in other cases the non-parametric Mann–Whitney test was applied. All tests were two-sided. A *p* < 0.05 was considered statistically significant.

## Results

There were no early or late deaths, and no early adverse events. All children were discharged within seven to 10 days after surgery. None of the patients in group 1 underwent reoperation or re-intervention in the first 12 months, while one patient in group 2 required surgical replacement of the conduit (see below). Compared to their preoperative functional class (five NYHA class I, six NYHA class II and one NYHA class III), all 12 patients in group 1 were in NYHA class I at 12 months. In group 2 5 patients were in NYHA class I and one in class 2 prior to surgery; all were in NYHA class I at 12 months of follow-up, see Table 1 for patient overview.

**Table 1:** Summary of patient outcomes.

**Patient ID**	**Mean gradient [mmHg]**			**PI severity**			**RVOT diameter [mm]**			**RV diastolic area [cm**^****2****^**]**			**NYHA class**	
	**7D**	**6M**	**12M**	**7D**	**6M**	**12M**	**7D**	**6M**	**12M**	**7D**	**6M**	**12M**	**Baseline**	**12M**
**GROUP 1**														
1	14	11	10	Mild	Trace	Mod	16	15	14	8.4	9.0	10.2	III	I
2	12	15	25	Trace	Mod	Mod	15	18	17	18.1	19.1	23.8	II	I
3	10	7	7	Mild	Mild	Mild	15	13	15	11.4	10.6	14.4	I	I
4	16	14	20	Trace	Mild	Sev	15	14	14	9.1	9.7	12.8	II	I
5	10	9	10	None	Mild	Mod	17	14	14	15.6	13.2	17.6	II	I
6	26	21	19	Trace	Mod	Mod	15	18	19	17.8	22.9	^*^	II	I
7	29	27	40	Mild	Mod	Mod	17	19	22	20.3	24.1	24.7	II	I
8	11	20	29	Trace	Mild	Mod	17	15	15	17.2	16.9	20.3	I	I
9	13	30	23	Mild	Sev	Sev	17	14	15	11.5	11.1	11.9	I	I
10	23	20	42	Mild	Mod	Sev	17	14	16	11.6	14.0	14.0	I	I
11	14	18	18	Mild	Sev	Sev	13	16	17	^*^	^*^	16.6	I	I
12	39	64	80	Mild	Sev	Sev	^*^	^*^	22	25.9	35.4	32.9	II	I
**GROUP 2**														
13	20	19	16	Mild	Mild	Trace	16	16	16	12.3	13.1	13.8	II	^*^
14	10	15	20	None	None	Mod	19	20	19	19.4	19.9	23.1	I	I
15	8	14	6	Mild	Trace	Mild	18	18	17	12.2	^*^	8.0	I	I
16	10	9	10	Trace	Trace	Mild	16	17	15	10.5	14.8	11.7	I	I
17	31	22	27	Mild	Mild	Mild	15	18	18	^*^	18.4	18.0	I	I
18	10	20	^*^	Mild	Mild	^*^	18	^*^	^*^	^*^	^*^	^*^	I	^*^

*7D, 7 days post-surgery; 6M, 6 months; 12M, 12 months; PI, Pulmonary Insufficiency, classified as None, Trace, Mild, Moderate (Mod) or Severe (Sev). NYHA, New York Heart Association (NYHA) classification of limitation during physical activity: I -No limitation II -Slight limitation III -Marked limitation IV -Unable without discomfort*.

* *Data not available*.

### Conduit Function

#### Gradient

**Figure 2:**
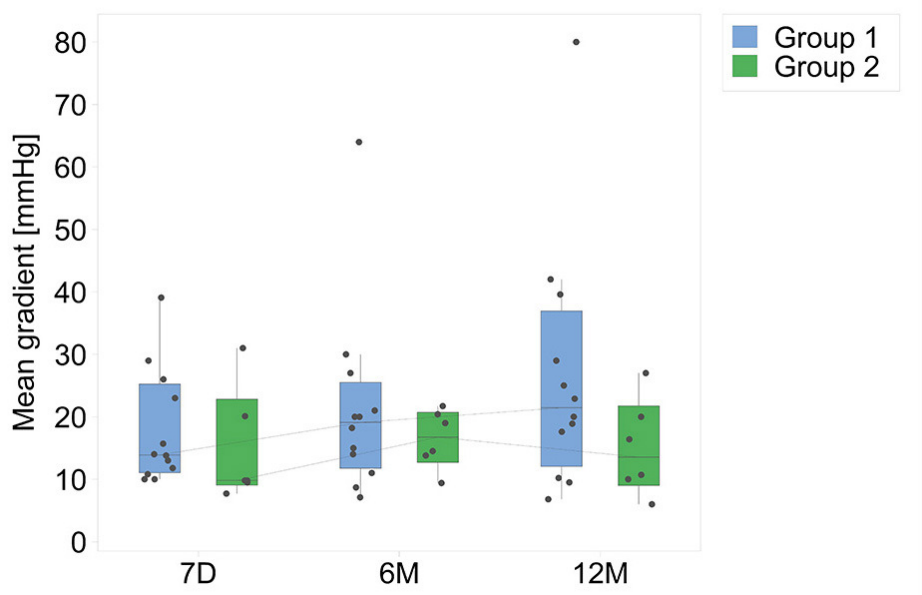
Mean Doppler gradients (mm Hg), for both groups, from hospital discharge up to 12 months. D7, 7 days post-surgery; 6M, 6 months; 12M, 12 months.

In nine of the 12 patients in group 1, the peak Doppler gradient on echocardiography remained below 40 mm Hg at the 12 month follow-up (mean gradient of 26.9 ± 20 mm Hg at 12 months for group 1). Of the three children with peak gradients of >40 mm Hg, two patients had undergone concomitant patch augmentation of the branch pulmonary arteries at the time of XPV implantation. In both of them the residual gradient could be demonstrated to be downstream to the distal anastomotic site of the conduit. One patient showed echocardiographic evidence of kinking of the conduit at the proximal anastomosis, which was considered to be a surgical technical issue, and had a peak Doppler gradient of >40 mm Hg at follow-up. In group 2, five of the six patients had a peak gradient of <40 mm Hg at 12 months (mean gradient of 15.9 ± 16.4 mm Hg at 12 months for group 2). The serial changes in mean Doppler echocardiographic gradient up to 12 months of follow-up are shown in Figure 2.

**Figure 3:**
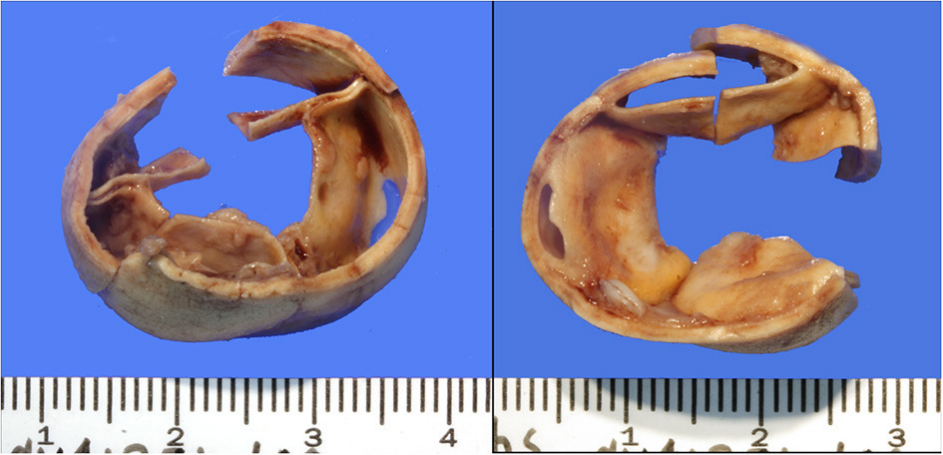
Downstream (left hand panel, looking from the side of the pulmonary trunk, and upstream (looking from the RV side, right hand panel) of the explanted conduit. The leaflets are well-preserved, with mild commissural fusion at the base. The lumen of the conduit is also smooth, without evidence of excessive tissue proliferation or potential obstruction.

One child in group 2, with a diagnosis of tuberous sclerosis and multiple cardiac rhabdomyomas, had undergone balloon valvuloplasty of the aortic valve at 3 months and 7 months of age. At 10 months, the child underwent surgical excision of two rhabdomyomas at the base of the aortic leaflets, with concomitant left ventricular myectomy and aortic valve repair. At 4 years, a Ross operation was performed (replacement of the aortic root with use of the pulmonary autograft, and implantation of an 18 mm XPV conduit in the pulmonary position) for recurrent left ventricular outflow tract obstruction secondary to scarring of the area. At several time points the child had been noted to have a hyperinflammatory reaction to various interventions, and had been on immunosuppressive therapy, followed by oral ibuprofen prior to XPV implantation. Post XPV implant follow-up was clinically uneventful. At 6 months, a moderate gradient was seen on echocardiography (peak gradient 44 mm Hg; mean 20 mm Hg) at the site of the proximal anastomosis. These findings were reconfirmed at 9 months (moderate RVOT obstruction and mild PI). Nine days after the 9 month follow-up the patient presented with acute abdominal discomfort. Echocardiographic examination at that admission demonstrated severe conduit stenosis, and right ventricular dysfunction. The XPV conduit was explanted and a Contegra graft implanted. At surgery there was severe stenosis at the proximal and distal suture lines. Histological examination of the explanted XPV conduit showed organized fibrin on the luminal surface, with minimal inflammation and no neovascularization. There was intimal scarring at the proximal and distal anastomotic sites, confirming past findings for this patient. The valve leaflets were intact, with evidence of mild commissural fusion (Figure 3). Small thrombi were seen in two of the three valve sinuses. Despite the dramatic clinical course with deterioration within 9 days following an uneventful clinical and echocardiographic examination, these findings were attributed to a hyperinflammatory response which might be peculiar to tuberous sclerosis (17). The current Contegra conduit has also experienced premature narrowing prompting the use of immune modulating medication. This type of patient will require careful consideration prior to XPV implants in the future.

#### Diameter

**Figure 4:**
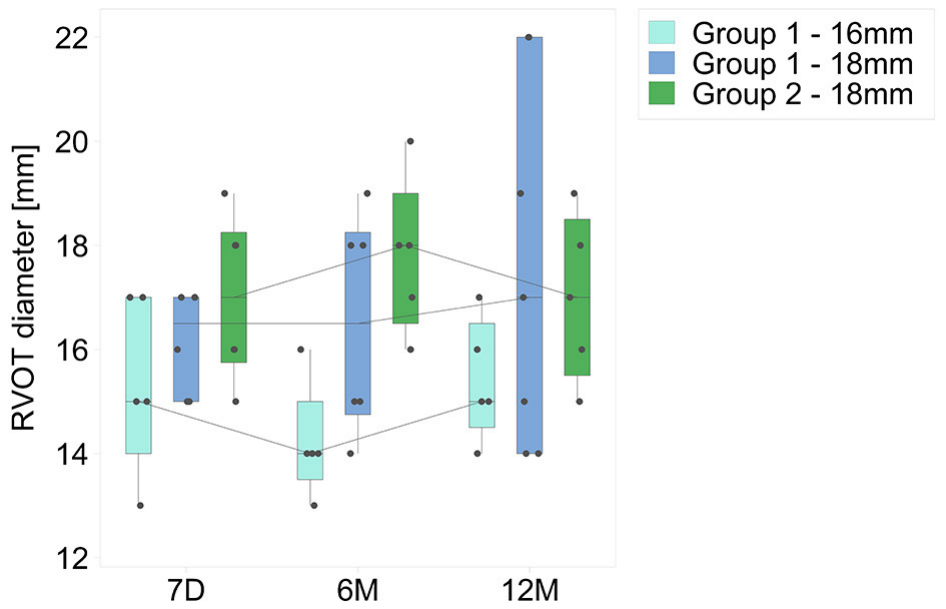
Serial changes in minimum conduit diameters (mm) at follow-up, for both groups. Time intervals are as for Figure 1.

There was a non-significant change in conduit diameter during follow-up in both groups. None of the conduits demonstrated aneurysm formation, or stenosis within the conduit outside of the suture lines, over time. Only one patient in group 2 (see description above) developed suture line stenosis at follow-up, possibly attributable to his co-morbid condition (tuberous sclerosis). Serial changes in RVOT diameter are shown in Figure 4.

#### Pulmonary Regurgitation

**Figure 5:**
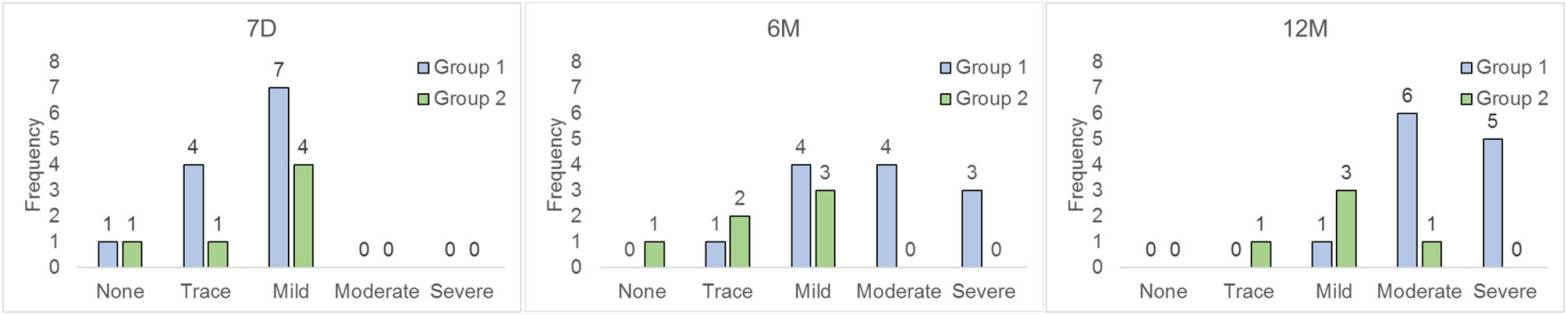
Frequency histogram of PI for both groups. Time intervals are as for Figure 1.

At hospital discharge, PI in group 1 was graded as “none to mild” in all patients. At 12 months, five patients had developed severe PI, and six had moderate regurgitation. Anatomical factors associated with the development of significant PI were analyzed. Two patients had significant residual gradients at the level of the branch pulmonary arteries, which could have contributed to the PI. Kinking of the conduit could have contributed to PI in one patient. The commonest mechanism of PI was prolapse (or flailing) of one of the three leaflets of the valve. This could be definitively identified in three patients, and was thought to be the potential mechanism in two others. Prolapse of a leaflet was first observed at the 6 month follow-up echocardiogram in three patients, and at the 12 month study in two others. In group 2, PI grade was “none or mild” in all six patients at hospital discharge. At 12 months, PI was mild in 4 patients, and moderate in one; in particular no patient demonstrated prolapse, or potential prolapse, of any of the valve leaflets. One patient required re-operation at 9 months (see above); the last echocardiogram prior to XPV replacement graded PI as “mild” in this child. Figure 5 is a frequency histogram of the grade of PI at discharge, 6 and 12 months follow-up.

#### Right Ventricular Size and Area

**Figure 6:**
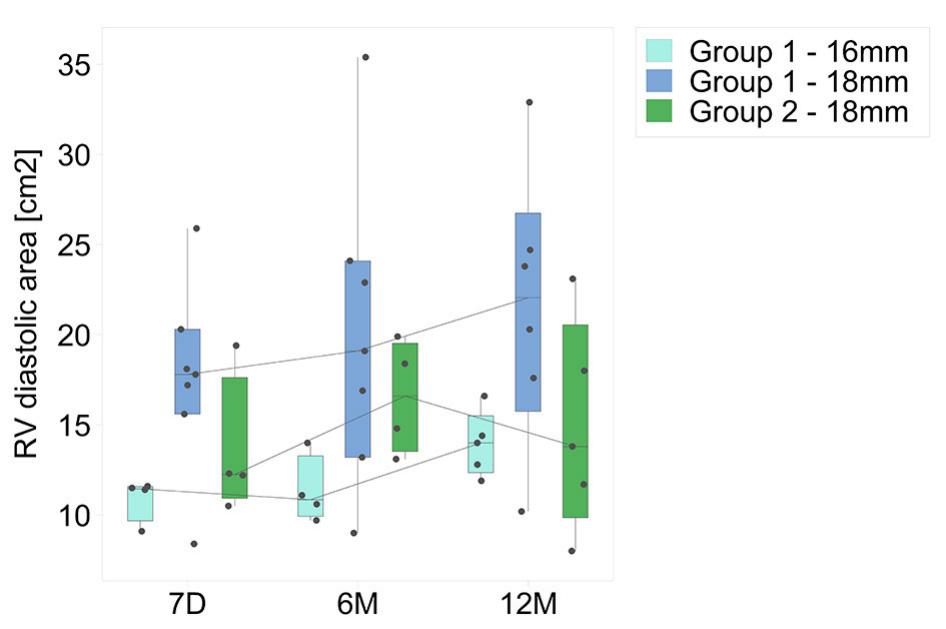
Calculated diastolic area for both groups. Time intervals are as for Figure 1.

Right ventricular end diastolic diameters (RVEDD) were measured serially at three separate sites; the basal segment at the level of the tricuspid valve annulus (basal), at mid-cavity level, and at the level of the RVOT, below the level of the conduit. The obtained diameters were used to calculate RV diastolic area (Figure 6). The only statistical difference between discharge and 12 months was found for the diastolic area of 16 mm group 1 valves (*p* = 0.02). No statistical differences were found for any RV parameters between groups (*p* > 0.05 in all cases). Also, on comparing group 1 patients with severe PI (*n* = 5) to those with less than severe PI (*n* = 7), none of the measurements showed any significant difference between the two subsets.

## Discussion

A wide variety of conduits are commercially available, but the perfect conduit unfortunately does not still exist. To date, the gold standard for RVOT reconstruction has been the allograft, harvested from cadavers. Valved pulmonary allografts were originally introduced by Ross et al. (18). They have been shown to be durable, particularly in adults. In the pediatric population, structural deterioration of the conduit leads to inevitable replacement (19–21). Particularly in young patients, conduit dysfunction, with resulting stenosis and valvar insufficiency, may be related to geometric distortion during the implant procedure. Wells et al. demonstrated that somatic outgrowth was not the major cause of allograft conduit failures in young patients. Failure was primarily due to conduit stenosis, with an additional 30% failure rate due to technical issues during surgery (22). Early allograft (or xenograft) conduit deterioration may be related to recipient immunological reactions, as has been suggested in several studies. Rajani et al. demonstrated histological evidence for rejection in allograft valves explanted from the right ventricular outflow tract in children. All valves implanted in infants had failed at >12 months following implant. All of them showed multiple foci of inflammation consisting of T lymphocytes (18). Similar findings were reported by Vogt and colleagues, from allograft material explanted from the right ventricular outflow tract (23). The limited availability of homografts has meant that allografts are increasingly used in clinical practice, such as the Contegra, a bovine jugular vein derived conduit. However, it is associated with the development of stenosis at the distal anastomosis, which may not be due to surgical technical issues. Histological study of the explanted conduits showed fibrointimal proliferation and peel formation at the distal anastomotic site, which obstructed the lumen (20). Meyns et al. demonstrated similar histologic findings in failed Contegra conduits, and suggested that the risk of development of distal stenosis did not change over time (24). Freedom from severe stenosis (defined as an echocardiographic peak gradient of >50 mm Hg) at the distal anastomosis was <50% at 24 months following conduit implantation. The authors hypothesized that chronic movement of the graft wall at the level of the anastomosis (a function related to the high degree of pliability of the Contegra graft) could cause repetitive trauma and be the ongoing trigger for fibrointimal peel formation. The FDA reported composite conduit failure rates at 1 year of 25% and 22% for the Contegra graft's FDA HDE submission (*n* = 237) and post-marketing study (*n* = 374), respectively, suggesting that alternatives are clearly required (22).

The XPV conduit has been designed to enable endogenous tissue restoration. No cell seeding, growth factors or other active ingredients were incorporated to guide the restoration process Nonetheless, animal studies with the XPV conduit showed complete coverage of the conduit wall with neointima was observed at 2 months after implantation. The leaflets themselves were functionally replaced with a well-vascularized collagen-rich connective tissue at between six and 12 months. Part of the promising performance of the XPV conduit may be attributed to the supramolecular RestoreX™ polymer platform. Its supramolecular UPy motif provides excellent mechanical strength at relatively low molecular weights, which facilitates the electrospinning process. In addition, the UPy motif provides a polymer platform, allowing tailored chemistry, such as a more slowly absorbing and flexible PC-UPy for the leaflets vs. a more stiff but faster absorbing PCL-UPy for the conduits. Finally, the XPV conduit appears to provide a favorable microenvironment for endogenous tissue restoration. Recent work using a subcutaneous rat model demonstrates that RestoreX™ trigger a dominant M2 macrophage response, which is a unique observation for synthetic materials, and is associated with a pro-remodeling tissue response. The mechanisms behind this predominant M2 response are subject of active investigation.

The handling properties of the XPV conduit are comparable to those of other synthetic conduits such as PTFE or Dacron, and conduits can be cut and refashioned during surgery. One patient in group 1 developed conduit stenosis due to a technical issue, with kinking of the conduit at implantation. This most likely also led to early onset of PI in this patient. One child in group 2, as described above, required early conduit replacement. The dramatic clinical presentation, with acute conduit stenosis and right ventricular failure 9 days after a seemingly normal routine follow-up 9 months post-XPV implantation, defies clear explanation. Tuberous sclerosis can be associated with overexpression of the complement pathways, and a hyperimmune reaction (16). The patient's current Contegra conduit has experienced the same process of early recurrence of stenosis. Importantly, apart from intimal scarring at the anastomotic lines, the lumen and leaflets of the explanted XPV-2 conduit did not exhibit any signs of degeneration.

An important finding in group 1 was the occurrence of severe PI in five of the 12 implanted conduits at the 12 months. The commonest identifiable mechanism for PI was prolapse of one of the valve cusps. A possible mechanism for leaflet prolapse and the development of significant PI could be early loss of mechanical integrity of the bioabsorbable foundation, and insufficient replacement by native material within this time frame, resulting in the leaflets being inadequately supported resulting in leaflet prolapse and loss of coaptation. This led to modification of leaflet design, as described, with improved thickness homogeneity of the leaflets. Bench testing demonstrated that the improved design had a significantly longer fatigue life, which is anticipated to provide more time for endogenous tissue to restore and take over functionality. First signs of improved clinical function are promising, showing a marked reduction in degree of severity of PI in group 2 at 12 months post-implant. It should be noted that this ability to learn and adapt based on clinical findings is an important and unique asset of the RestoreX™ platform, which may prove essential when expanding to larger patient groups and additional indications. For example, promising acute preclinical results using RestoreX for aortic valve restoration were previously reported (25).

### Potential Limitations

A relatively large number of centers have been involved in the study, with the possible result that surgical and technical issues were not addressed in a standardized way. The duration of follow-up is short, and hence graft growth to match somatic growth of the patient has not been demonstrated.

## Conclusions

The XPV conduit can be fashioned to any desired size, thickness, and degrees of leaflet coaptation, and made to specification. Degeneration of the conduit resulting in aneurysmal dilation has not been observed, and only one of 18 patients developed conduit stenosis, albeit under dramatic circumstances and for reasons that are unclear, but possibly related to an underlying inflammatory disorder. Improving leaflet strength has demonstrably proven that PI could be reduced in the short term as demonstrated by the XPV-2, demonstrating the potential of the RestoreX™ technology to adapt based on clinical learnings. Although patient numbers are small, and follow-up times are short, these results offer promising signs of the potential of this technology to change the treatment paradigm for children requiring RVOT reconstruction. The present outcome has allowed for an FDA clinical trial to be approved so that longer term follow-up of the current conduit design can be assessed.

## Data Availability Statement

The original contributions presented in the study are included in the article/supplementary materials, further inquiries can be directed to the corresponding author/s.

## Ethics Statement

The studies involving human participants were reviewed and approved by Regulatory Authority Approvals were obtained from each country. Infromed consent was obtained from the parents for all patients prior to the procedure (www.clinicaltrials.gov: NCT02700100 and NCT03022708). Written informed consent to participate in this study was provided by the participants' legal guardian/next of kin.

## Author Contributions

All authors listed have made a substantial, direct and intellectual contribution to the work, and approved it for publication.

## Conflict of Interest

MC is an employee of, or holds shares/options in Xeltis. FA directs an academic cardiovascular imaging core laboratory with institutional contracts to Xeltis (with no personal conflict of interest). The remaining authors declare that the research was conducted in the absence of any commercial or financial relationships that could be construed as a potential conflict of interest.

## Abbreviations

XPV: Xeltis pulmonary valve
RVOT: Right ventricular outflow tract
PI: Pulmonary insufficiency (regurgitation)
NYHA, class: New York Heart Association functional class.
